# Value of Assessing Autonomic Nervous Function by Heart Rate Variability and Heart Rate Turbulence in Hypertensive Patients

**DOI:** 10.1155/2018/4067601

**Published:** 2018-10-14

**Authors:** Yijun Yu, Yanling Xu, Mingjing Zhang, Yuting Wang, Wusong Zou, Ye Gu

**Affiliations:** Department of Cardiology, Wuhan Fourth Hospital, Puai Hospital Affiliated to Tongji Medical College, Huazhong University of Science and Technology, Wuhan 430030, China

## Abstract

**Purpose:**

To explore the relationship between blood pressure control and autonomic nervous function assessing by heart rate variability (HRV) and heart rate turbulence (HRT) in hypertensive patients.

**Methods:**

A total of 120 consecutive hypertensive patients and 80 nonhypertensive patients (N-HP group) were enrolled in this study. The hypertensive patients were divided into controlled blood pressure and uncontrolled blood pressure groups according to their blood pressure on admission. All subjects underwent 24-hour Holter monitoring. This study compared HRV and HRT in nonhypertensive and hypertensive patients and hypertensive patients with controlled and uncontrolled blood pressure. HRV parameters include square root of mean of the sum of squares of successive NN interval differences (rMSSD), number of successive NN intervals differing by > 50ms divided by the total number of successive NN intervals (pNN50), very low frequency (VLF) at frequency between 0.0033 and 0.04 Hz, low frequency (LF) at frequency between 0.04 and 0.15 Hz, and high frequency (HF) at frequency between 0.15 and 0.4 Hz. Turbulence slope (TS) belongs to HRT parameters.

**Results:**

TS, rMSSD, pNN50, VLF, LF, and HF values were significantly lower in the HP group than in the N-HP group. Multiple logistic regression analysis showed that reduced TS, rMSSD, pNN50, LF, and HF values were risk factors of hypertension. TS, rMSSD, pNN50, VLF, LF, and HF values were significantly lower in hypertensive patients with uncontrolled blood pressure than in hypertensive patients with controlled blood pressure. Multiple logistic regression analysis showed that reduced TS, rMSSD, pNN50, VLF, LF, and HF values were risk factors for uncontrolled blood pressure.

**Conclusions:**

This study indicates impaired autonomic nervous function in hypertensive patients, especially in hypertensive patients with uncontrolled blood pressure despite guideline recommended antihypertensive medications.

## 1. Introduction

 Hypertension is a major disease that damages people's health. Long-term hypertension could impair major organs such as heart, brain, kidneys, and blood vessels, which is related to considerable mortality [1]. Sympathetic overactivation and autonomous imbalance play important roles in the pathogenesis of hypertension. Heart rate variability (HRV) and heart rate turbulence (HRT) reflect the autonomic regulation of cardiac function. HRV is the response of autonomic nervous system to external environmental stimuli, and HRT is the response to autonomic nervous function triggered by endogenous ventricular premature beat. Abnormal HRV and HRT reflected autonomous imbalance and were related to worse cardiovascular outcome [2–5]. Abnormal HRV or HRT was demonstrated in hypertensive patients in previous studies [6–8]. However, there was scantly research on the relationship between HRV, HRT, and blood pressure control with hypertensive patients. The present study analyzed the HRV and HRT between nonhypertensive (N-HP) patients and hypertensive patients and between hypertensive patients with uncontrolled blood pressure and controlled blood pressure after hypertensive medication.

## 2. Materials and Methods 

### 2.1. Study Population

A total of 120 consecutive hospitalized hypertensive patients and 80 N-HP patients were included in this retrospective study from June 2016 to June 2018. The hypertensive patients were divided into controlled blood pressure (n=66) and uncontrolled blood pressure (n=54) groups according to their blood pressure on admission.

Patients with Diabetes Mellitus (DM), Acute Coronary Syndrome (ACS), valvular heart disease and known nonischemic cardiomyopathy, atrial fibrillation, atrial flutter, 2nd- or 3rd-degree atrioventricular block, and pacemaker implantation and patients without premature ventricular contraction (PVC) of 24-hour Holter monitoring were excluded. All hypertensive patients received antihypertensive medication. All patients gave informed consent for participation in this study, and the study protocol was approved by the ethical committees of Wuhan Fourth Hospital, Puai Hospital affiliated to Tongji Medical College, Huazhong University of Science and Technology, Wuhan, China.

### 2.2. HRV Analysis

All participants underwent 24-hour Holter monitoring (GE MARS Software and Seer Light recording box). Quantitative HRV analysis was performed according to the guidelines of the European Society of Cardiology and the North American Society of Pacing and Electrophysiology [9]. HRV parameters were derived from Holter monitoring including time domain and frequency domain. The following four time domain and four frequency domain indexes were analyzed: standard deviation of NN intervals (SDNN), standard deviation of all 5-minute average NN intervals (SDANN), square root of mean of the sum of squares of successive NN interval differences (rMSSD), number of successive NN intervals differing by > 50ms divided by the total number of successive NN intervals (pNN50), very low frequency (VLF) at frequency between 0.0033 and 0.04 Hz, low frequency (LF) at frequency between 0.04 and 0.15 Hz, high frequency (HF) at frequency between 0.15 and 0.4 Hz, and low frequency/high frequency ratio (LF/HF).

### 2.3. HRT Analysis

HRT parameters were also derived from Holter monitoring including turbulence onset (TO) and turbulence slope (TS). TO was the amount of sinus acceleration following a PVC. TO was expressed as a percentage and was calculated with the following formula: TO (%) = 100 × [(RR1 + RR2) − (RR−1 +RR−2)]/(RR−1 + RR−2), where RR1 and RR2 were the first and second sinus RR intervals after the PVC, and RR−1 and RR−2 were the first and second sinus intervals preceding the PVC. TO value < 0% indicated early sinus acceleration and was considered normal. TO ≥ 0% indicated that normal sinus heart rate acceleration phenomenon after PVC disappeared and was described as abnormal [5]. TS was late deceleration phenomenon of sinus rhythm after PVC following the sinus acceleration. TS was defined as the maximum regression slope measured on any 5- consecutive sinus beats within the first 15-sinus intervals after a PVC. TS could not be calculated when there were fewer than 15-sinus beats after the PVC. TS value > 2.5 ms/RR interval indicated the normal expected late deceleration. TS ≤ 2.5 ms/RR interval is described as abnormal [5]. TO and TS were computed as an average of the responses to all PVC on Holter record.

### 2.4. Statistical Analysis

Continuous data were presented as mean ± standard deviation (SD). Normal distribution of continuous variables was performed by Kolmogorov-Smirnov test. Continuous variables with normal distribution were assessed by Student's* t*-test. Nonnormal distribution data were tested by two-tailed Mann–Whitney* U* test. The chi-square test was used to compare categorical variables as percentages. The risk factors for hypertension were determined by multivariate logistic regression model after adjusting for age, gender, and beta-blockers use. Spearman correlation analysis of the hypertensive patients was performed between HRV and HRT.* P* values less than 0.05 were considered statistically significant. Statistical analyses were performed using IBM SPSS (version 22.0) for Windows (SPSS).

## 3. Results 

### 3.1. Clinical Features of Patients in N-HP and HP Groups

**Figure 1:**
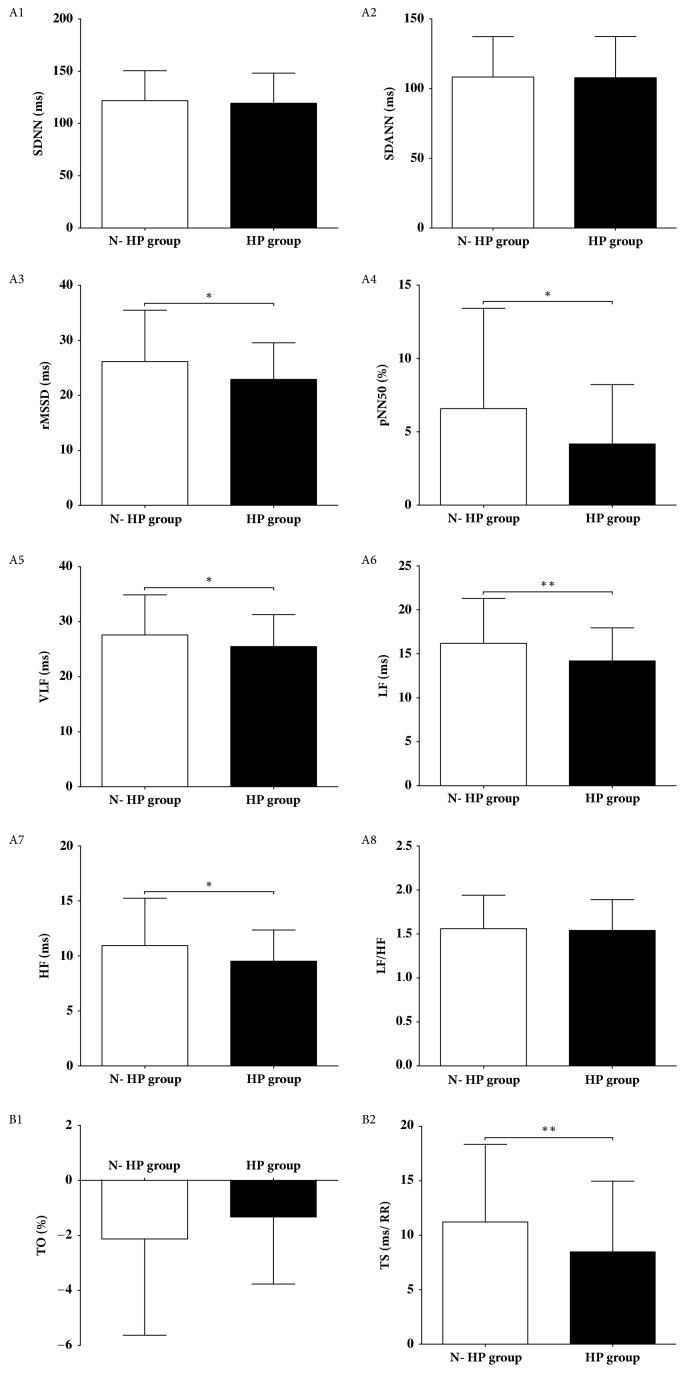
HRV and HRT analysis of N-HP and HP groups; *∗P*<0.05; *∗∗P*<0.01. HRV, heart rate variability; HRT, heart rate turbulence; N-HP, nonhypertensive; HP, hypertensive; SDNN, standard deviation of NN intervals; SDANN, standard deviation of all 5-minute average NN intervals; rMSSD, square root of mean of the sum of squares of successive NN interval differences; pNN50, number of successive NN intervals differing by >50ms divided by the total number of successive NN intervals; VLF, very low frequency; LF, low frequency; HF, high frequency; TO, turbulence onset; TS, turbulence slope.

BMI, triglyceride level, interventricular septum (IVS) thickness, and incidence of stable CAD were significantly higher in the HP group compared to the N-HP group. Blood pressure on admission was significantly higher in the HP group compared to the N-HP group. The proportions of beta-blockers and diuretics uses were higher in the HP group than in the N-HP group (Table 1). TS, rMSSD, pNN50, VLF, LF, and HF values were significantly lower in the HP group than in the N-HP group (Figure 1). Multiple regression analysis showed that history of stable CAD, higher BMI, and reduced TS, rMSSD, pNN50, LF, and HF values were risk factors of hypertension after adjusting for gender, age, and beta-blockers use (Table 2).

### 3.2. Clinical Features of Hypertensive Patients with Controlled and Uncontrolled Blood Pressure

**Figure 2:**
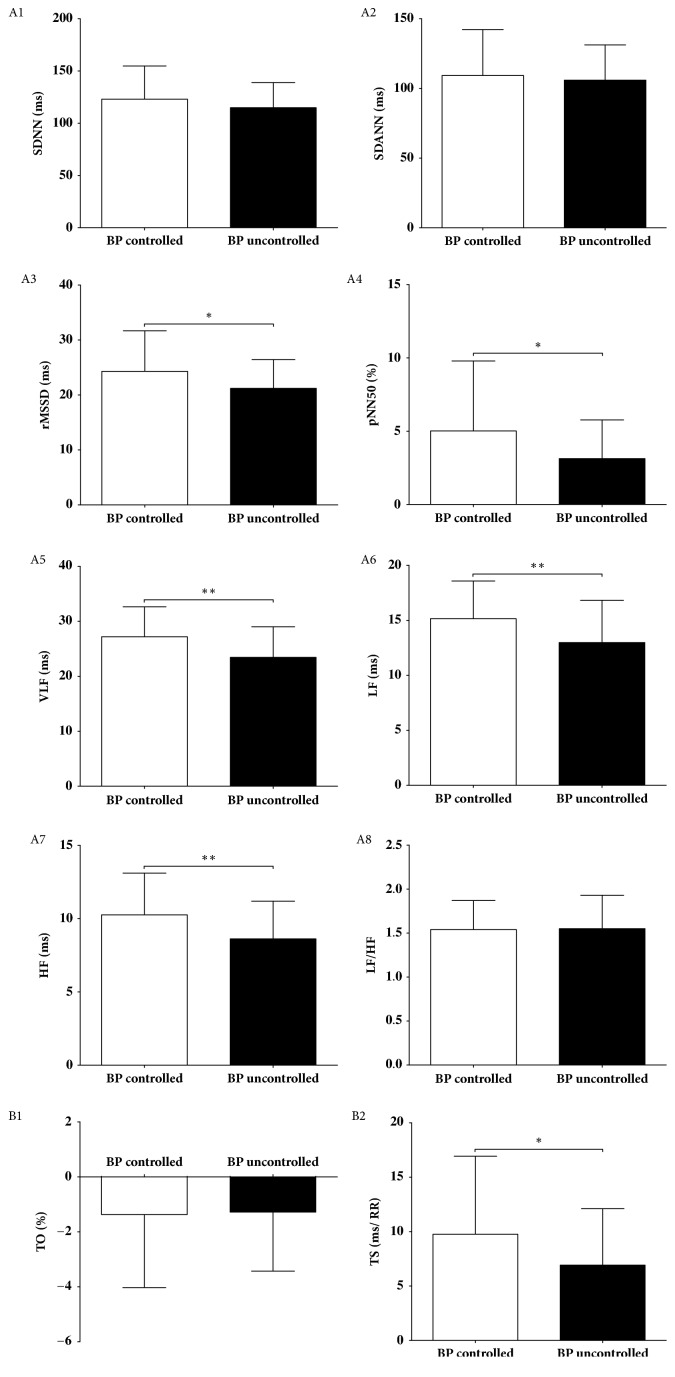
HRV and HRT analysis of BP controlled and BP uncontrolled groups, *∗P*<0.05; *∗∗P*<0.01. HRV, heart rate variability; HRT, heart rate turbulence; BP, blood pressure; SDNN, standard deviation of NN intervals; SDANN, standard deviation of all 5-minute average NN intervals; rMSSD, square root of mean of the sum of squares of successive NN interval differences; pNN50, number of successive NN intervals differing by >50ms divided by the total number of successive NN intervals; VLF, very low frequency; LF, low frequency; HF, high frequency; TO, turbulence onset; TS, turbulence slope.

The percentage of hypertensive patients receiving combined antihypertensive drug therapy was significantly higher and percentage of patients treating with monotherapy was significantly lower in hypertensive patients with uncontrolled blood pressure compared to hypertensive patients with controlled blood pressure (Table 3). TS, rMSSD, pNN50, VLF, LF, and HF values were significantly lower in hypertensive patients with uncontrolled blood pressure compared to hypertensive patients with controlled blood pressure (Figure 2). Multiple logistic regression analysis showed that reduced TS, rMSSD, pNN50, VLF, LF, and HF values were risk factors for blood pressure control after adjusting for age, gender, and beta-blockers use (Table 4).

### 3.3. Spearman Correlation of HRV and HRT for Hypertensive Patients

Spearman correlation analysis of the hypertensive patients showed that LF and LF/HF were negatively correlated with TO, while SDNN, SDANN, rMSSD, PNN50, VLF, LF, and HF were positively correlated with TS (Table 5).

## 4. Discussion

The present study found that TS, rMSSD, pNN50, VLF, LF, and HF values were significantly lower in hypertensive patients compared to N-HP patients, and TS, rMSSD, pNN50, VLF, LF, and HF values were significantly lower in hypertensive patients with uncontrolled blood pressure compared to hypertensive patients with controlled blood pressure. Our study results thus indicate impaired autonomic nervous function in hypertensive patients, especially in hypertensive patients with uncontrolled blood pressure despite guideline recommended antihypertensive medications. To the best of our knowledge, this is the first study describing the association between autonomic nervous function, evaluated by HRV and HRT changes, and blood pressure control in hypertensive patients.

### 4.1. Reduced HRV and HRT in Hypertensive Patients

HRV and HRT changes could reflect sympathetic and vagal function in hypertensive patients. HRV reflects the fluctuation of heart rate as time changes in response to external environmental stimulation; HRV changes were related to various cardiovascular diseases [3]. HRT reflects the start acceleration and the late deceleration of the heart rate after ventricular premature contraction and refers the endogenous stimulus triggered pressure reflex regulation and could also be used to evaluate the balance and coordination of the cardiac autonomic nervous system [5]. Combined analysis with HRV and HRT parameters makes it possible to comprehensively evaluate the autonomic nervous system regulation and response status to internal and external stimuli in hypertensive patients. Pal and colleagues [7] demonstrated enhanced sympathetic nerve activity and inhibited vagal activity in prehypertensive patients and found that the vagal inhibition was more prominent than sympathetic overactivity in hypertensive patients. Erdem [10] explored the relationship between autonomic nervous regulation and blood pressure in prehypertensive patients and found that TO was significantly higher and TS was significantly lower in nondipper blood pressure group than in dipper blood pressure group, hinting at impaired autonomous balance in prehypertensive patients with nondipper blood pressure. Another study [11] reported that heart rate was increased and HRV was decreased in patients with refractory hypertension, suggesting that overactivation of the sympathetic nervous system might play an important role in patients with refractory hypertension. In a previous study [12], we demonstrated significant differences on autonomous balance in hypertensive patients with controlled and uncontrolled blood pressure. The present study showed that TS (reflecting vagus function triggered by endogenous ventricular premature beat [13]), rMSSD (reflecting vagus function by external environmental stimuli [14]), pNN50 (reflecting vagus function by external environmental stimuli [14]), VLF (reflecting sympathetic activity by external environmental stimuli [15]), LF (reflecting balance of sympathetic and vagal activity [14]), and HF (reflecting vagus function by external environmental stimuli [14]) values were significantly lower in hypertensive patients compared to N-HP patients, and TS, rMSSD, pNN50, VLF, LF, and HF values were also significantly lower in hypertensive patients with uncontrolled blood pressure compared to hypertensive patients with controlled blood pressure. This novel finding demonstrated that autonomic nervous function was impaired in hypertensive patients compared to N-HP patients. Moreover, autonomic nervous function damage was more severe in hypertensive patients with uncontrolled blood pressure than in hypertensive patients with controlled blood pressure, as expressed by sympathetic overactivity and vagal withdrawal triggered by external environmental stimuli and vagal withdrawal triggered by endogenous ventricular premature beat. In our study, the percentage of hypertensive patients receiving combined antihypertensive drug therapy was significantly higher and percentage of patients treated with monotherapy was significantly lower in hypertensive patients with uncontrolled blood pressure compared to hypertensive patients with controlled blood pressure, indicating that the uncontrolled blood pressure observed in our patient cohort is probably not due to the insufficient hypertensive medication; future studies are warranted to explore the role of the more severe autonomous function impairment in hypertensive patients with uncontrolled blood pressure despite the treatment of guideline recommended antihypertensive medications and to see if options targeting the autonomic nervous function might help the blood pressure control on top of combined antihypertensive therapy [16].

Previous studies found that DM and beta-blockers use might affect the HRV [15, 17]. Patients with DM were thus excluded in our study. Results of logistic regression analysis showed that reduced TS, rMSSD, pNN50, VLF, LF, and HF values were risk factors for uncontrolled blood pressure after adjusting for age, gender, and beta-blockers use. Therefore, the difference in HRV and HRT values between the uncontrolled and controlled blood pressure groups was unlikely induced by beta-blockers use.

HRV mainly reflected the interaction between neural modulatory and sinus node function, while HRT could be considered as parameter reflecting the physiological response to endogenous stimulus. Spearman correlation analysis between HRV and HRT showed that LF and LF/HF were negatively correlated with TO, and SDNN, SDANN, rMSSD, PNN50, VLF, LF, and HF were positively correlated with TS, which suggested the close correlation between HRV and TS, and HRV and HRT could be considered as complementary parameters reflecting autonomic nervous function change.

### 4.2. Clinical Implications

Impaired autonomic function played an important role in the pathogenesis of hypertension. Long-term sympathetic excitation might lead to left ventricular remodeling and atherosclerosis. Poreba et al. [8] found that TO was significantly higher and TS was significantly lower in hypertensive patients with left ventricular hypertrophy than in hypertensive patients without left ventricular hypertrophy. Therefore, the detection of autonomic nervous function in hypertensive patients might be useful in predicting the target organ damage in hypertensive patients. Abnormal HRV and HRT in hypertensive patients might suggest the presence of autonomic nervous system dysfunction. The present results found abnormal HRV and HRT in hypertensive patients, especially in hypertensive patients with uncontrolled blood pressure. It is thus clinically important to monitor HRV and HRT during antihypertensive therapy, aiming to improve the autonomic nervous system function in hypertensive patients, which might reduce the incidence of target organ damage and improve the prognosis of hypertensive patients.

### 4.3. Study Limitations

There were some limitations in this study. First, this was a retrospective single-center clinical study with a small number of patients. Our results need to be confirmed by a multicenter prospective clinical study with larger patient cohort to explore the impact of autonomic nervous dysfunction on prognosis of hypertensive patients. Second, HRV and HRT evaluation was not suitable to hypertensive patients with nonsinus rhythm such as atrial fibrillation, atrial flutter or pacemaker implantation, or 2nd- or 3rd-degree atrioventricular block and without PVC on Holter monitoring. Third, we did not quantify cardiac remodeling parameters including left ventricular posterior wall thickness and diastolic function parameters as E/A and E/e' in this patient cohort. Finally, this study did not analyze potential impact of the disease stage as well as the duration of antihypertensive medication on HRV and HRT because many elderly patients in this patient cohort could not provide us with the inquired data. Above study limitations should be considered when interpreting results demonstrated in this study.

## 5. Conclusions

The present study shows that autonomic nervous dysfunction, as expressed by reduced HRV and HRT, exists in hypertensive patients, especially in hypertensive patients with uncontrolled blood pressure. Monitoring HRV and HRT parameters, which jointly reflect autonomic nervous system's regulation and response to internal and external stimuli, might be helpful to evaluate the autonomic nervous function status of the patients and supply useful information to optimize therapeutic efficacy aiming to improve autonomic nervous function balance for hypertensive patients. Future studies are warranted to explore if targeting the autonomic nervous function on top of antihypertensive medication might obtain better clinical efficacy on blood pressure control for patients with refractory hypertension.

## Figures and Tables

**Table 1:** Clinical characteristic of N-HP group and HP group.

	N-HP group	HP group	*P* value
	(n=80)	(n=120)	
Age (yr)	56.66±6.62	58.05±7.55	0.183
Male gender (n, %)	39/80 (48.5%)	54/120 (45.0%)	0.602
BMI (kg/m^2^)	23.60±2.78	25.20±3.29	<0.0001
Smoker (n, %)	21/80 (26.3%)	40/120 (33.3%)	0.286
Stable CAD (n, %)	12/80 (15.0%)	37/120 (30.8%)	0.011
Dyslipidemia (n, %)	64/80 (80.0%)	105/120 (87.5%)	0.151
Systolic blood pressure (mmHg)	118.50±11.75	134.98±14.95	<0.0001
Diastolic blood pressure (mmHg)	76.10±7.48	81.98±10.15	<0.0001
Heart rate (bpm)	74.18±6.62	73.11±8.16	0.307
Creatinine (*μ*M)	67.44±16.33	67.34±14.63	0.839
CHOL (mM)	4.67±0.91	4.83±1.00	0.228
TG (mM)	1.59±0.98	2.13±2.10	0.002
LDL-c (mM)	2.96±0.83	2.92±0.82	0.742
HDL-c (mM)	1.09±0.25	1.10±0.27	0.848
Ejection fraction (%)	61.61±4.95	61.91±5.17	0.657
LVEDd (cm)	4.39±0.39	4.40±0.44	0.843
IVS (cm)	0.93±0.12	0.99±0.19	0.010
Medication			
Bata-blockers use (n, %)	23/80 (28.8%)	60/120 (50.0%)	0.003
Diuretics use (n, %)	0/80 (0.0%)	12/120 (10.0%)	0.009

N-HP, nonhypertensive; HP, hypertensive; BMI, body mass index; CAD, coronary artery disease; CHOL, cholesterol; TG, triglyceride; LDL-c, low-density lipoprotein cholesterol; HDL-c, high-density lipoprotein cholesterol; LVEDd, left ventricular end diastolic diameter; IVS, interventricular septum.

**Table 2:** Multivariate logistic regression results for risk of hypertension.

	B	S.E	Wald	*P* value	Exp	95% CI lower limit	95% CI upper limit
BMI	0.196	0.053	13.788	0.000	1.217	1.097	1.350
Stable CAD	0.832	0.395	4.431	0.035	2.297	1.059	4.982
TG	0.413	0.163	6.387	0.011	1.511	1.097	2.082
rMSSD (ms)	0.044	0.020	4.804	0.028	1.045	1.005	1.086
pNN50 (%)	0.070	0.031	5.249	0.022	1.073	1.010	1.139
VLF (ms)	0.039	0.024	2.716	0.099	1.041	0.993	1.091
LF (ms)	0.100	0.037	7.187	0.007	1.105	1.027	1.189
HF (ms)	0.096	0.046	4.356	0.037	1.100	1.006	1.203
TS (ms/ RR)	0.055	0.023	5.684	0.017	1.057	1.010	1.106

BMI, body mass index; CAD, coronary artery disease; TG, triglyceride; rMSSD, square root of mean of the sum of squares of successive NN interval differences; pNN50, number of successive NN intervals differing by >50ms divided by the total number of successive NN intervals; VLF, very low frequency; LF, low frequency; HF, high frequency; TS, turbulence slope.

**Table 3:** Clinical characteristics of hypertensive patients with controlled blood pressure group and uncontrolled blood pressure group.

	BP controlled group	BP uncontrolled group	*P* value
	(n=66)	(n=54)	
Age (yr)	57.03±6.81	59.30±8.26	0.109
Male gender (n, %)	27/66 (40.9%)	27/54 (50.0%)	0.319
BMI (kg/m^2^)	25.19±3.34	25.22±3.25	0.954
Smoker (n, %)	20/66 (30.3%)	20/54 (37.0%)	0.436
Stable CAD (n, %)	18/66 (27.3%)	19/54 (35.1%)	0.350
Dyslipidemia (n, %)	58/66 (87.9%)	47/54 (87.0%)	0.890
SBP (mmHg)	124.97±9.72	147.20±10.45	<0.0001
DBP (mmHg)	78.02±7.43	86.82±10.97	0.000
Heart rate (bpm)	72.35±8.27	74.03±8.00	0.364
Creatinine (*μ*M)	67.14±14.87	67.59±14.46	0.867
CHOL (mM)	4.83±0.93	4.84±1.08	0.945
TG (mM)	2.00±1.49	2.29±2.68	0.663
LDL-c (mM)	2.97±0.81	2.86±0.84	0.472
HDL-c (mM)	1.11±0.25	1.09±0.30	0.436
Ejection fraction (%)	62.33±4.78	61.39±5.60	0.321
LVEDd (cm)	4.40±0.48	4.41±0.39	0.907
IVS (cm)	1.00±0.15	1.00±0.22	0.891
Medication			
Bata-blockers (n, %)	32/66 (48.5%)	28/54 (51.9%)	0.714
ACEI (n, %)	14/66 (21.2%)	12/54 (22.2%)	0.894
ARBs (n, %)	19/66 (28.8%)	23/54 (42.6%)	0.115
CCB (n, %)	37/66 (56.1%)	39/54 (72.2%)	0.068
Diuretics (n, %)	6/66 (9.1%)	6/54 (11.1%)	0.714
Categories of drugs			0.021
Monotherapy (n,%)	32/66 (48.5%)	15/54 (27.7%)	
≥Two-drug therapy (n, %)	34/66 (51.5%)	39/54 (72.2%)	

BP, blood pressure; BMI, body mass index; CAD, coronary artery disease; SBP, systolic blood pressure; DBP, diastolic blood pressure; CHOL, cholesterol; TG, triglyceride; LDL-c, low-density lipoprotein cholesterol; HDL-c, high-density lipoprotein cholesterol; LVEDd, left ventricular end diastolic diameter; IVS, interventricular septum; ACEI, angiotensin-converting enzyme inhibitor; ARBs, angiotensin receptor blocker; CCB, calcium channel blocker.

**Table 4:** Multivariate logistic regression results for risk of uncontrolled blood pressure.

	B	S.E	Wald	*P* value	Exp	95% CI lower limit	95% CI upper limit
rMSSD (ms)	0.073	0.032	5.363	0.021	1.075	1.011	1.144
pNN50 (%)	0.131	0.058	5.130	0.024	1.140	1.017	1.277
VLF (ms)	0.128	0.038	11.358	0.001	1.136	1.055	1.225
LF (ms)	0.166	0.058	8.245	0.004	1.181	1.054	1.321
HF (ms)	0.213	0.076	7.957	0.005	1.238	1.067	1.435
TS (ms/ RR)	0.071	0.034	4.453	0.035	1.073	1.005	1.147

rMSSD, square root of mean of the sum of squares of successive NN interval differences; pNN50, number of successive NN intervals differing by >50ms divided by the total number of successive NN intervals; VLF, very low frequency; LF, low frequency; HF, high frequency; TS, turbulence slope.

**Table 5:** Spearman correlation analysis of HRV and HRT in HP patients.

	TO		TS	
	*r* value	*P* value	*r* value	*P* value
SDNN	-0.008	0.930	0.298	0.001
SDANN	0.023	0.800	0.260	0.004
rMSSD	0.006	0.945	0.292	0.001
pNN50	-0.012	0.895	0.228	0.012
VLF	-0.143	0.120	0.438	<0.0001
LF	-0.237	0.009	0.441	<0.0001
HF	-0.027	0.767	0.343	<0.0001
LF/HF	-0.241	0.008	0.095	0.301

HRV, heart rate variability; HRT, heart rate turbulence; HP, hypertensive; SDNN, standard deviation of NN intervals; SDANN, standard deviation of all 5-minute average NN intervals; rMSSD, square root of mean of the sum of squares of successive NN interval differences; pNN50, number of successive NN intervals differing by >50ms divided by the total number of successive NN intervals; VLF, very low frequency; LF, low frequency; HF, high frequency; TO, turbulence onset; TS, turbulence slope.

## Data Availability

The data used to support the findings of this study are available from the corresponding author upon request.
